# An Account of the Successful Treatment of a Case in Which the Brachial Artery Was Divided

**Published:** 1793

**Authors:** William Adair

**Affiliations:** Surgeon General to the Garrison of Gibraltar.


II. *An Account of the successful Treatment of a Case in which the Brachial Artery was divided. By* William Adair, *Esq. Surgeon General to the garrison of Gibraltar. Communicated in a Letter to* Everard Home, *Esq. F. R. S. and by him to Dr.* Simmons.

ON the 29th of July, 1792, a Genoese, about twenty years of age, by occupation a porter, was, in an affray, stabbed by a Portuguese in several parts of his body, with a jagged-edged long knife or poignard; but none of the wounds were of any consequence, excepting one across the Biceps muscle, just above the usual place of bleeding in the left arm. An empirical practitioner dressed the wounds; and observing a very considerable hæmorrhage from that in the left arm, filled it with lint and flour, and bound it up so tight as to stop the bleeding; but in doing this he had at the same time stopped all circulation in the arm below. Mr. Kidston, surgeon's mate of the sixty-first regiment, happened, about two hours after, to see the man, and finding the extremity of the limb benumbed, cold, and void of circulation, he removed the bandage, and a torrent of blood immediately gushed from the wound. He then made a compress on the artery below the axilla, by means of a tourniquet, and sent for me.

It was obvious, that in this case the brachial artery was divided, and that considering the torpid state of the arm, either amputation, or an operation the same as for an aneurism, must be immediately performed. On account of the poor man's situation, having his subsistence to earn by his labour, I determined upon the latter. Accordingly in presence of Mr. Patrick, surgeon of the sixty-first regiment, and Mr. Kidston, his mate, (from both of whom I received very great assistance) I began a piece of very intricate dissection, and took up all the divided blood-vessels completely; but in doing this, I think it right to remark, we lay under very great disadvantages; for the circulation in the arm having been suspended upwards of two hours, it became necessary to operate without delay; and we were obliged to operate in a small room, with bad light, occasioned partly by the badness of the candles, and partly by the heat of the weather and the number of persons that crowded about us, and rendered the air of the room impure. We had no place but the bed to lay lay the patient upon, and there was hardly ſpace ſufficient for us to move round it. To theſe inconveniences muſt be added another ariſing from the wound itſelf, which had been made with a jagged inſtrument, and penetrated deep into the hollow of the cubit, forming a mangled opening upon the lower edge of the biceps, ſo that notwithſtanding this opening was made free and large, I found it impracticable to ſeparate diſtinctly the blood-veſſels by diſſection. I therefore was obliged to take up by the needle every veſſel from which, upon ſlackening the tourniquet, we ſaw the blood iſſue; and it was not till we had made ſix ligatures, that we ſeemed to be ſecure againſt the danger of hæmorrhage, and in making theſe I have not a doubt that the ſheaths and aponeuroſes of tendons and muſcles, and likewiſe nerves, were included.

From the beginning of the operation to the final dreſſing of the arm, took us up an hour and three quarters. The patient behaved with great firmneſs during the whole of this period. It is now twelve weeks ſince the operation, and the wound is almoſt completely healed. No interruption to the progreſs of the cure has exiſted for a day, and he has had occaſion for no other medicines than a ſmall quantity of bark, and ſome vitriolic acid, during the whole of the treatment.

I was in continual apprehenſion from the ſeaſon of the year, and the almoſt abſolute certainty of tendinous parts having been taken up by the ligatures, that he would have had a locked jaw; but happily he eſcaped this ſymptom, which in this climate, and at this ſeaſon, has occurred with more frequency, after wounds, than in any other part of the world I have ever been in, and even from the ſlighteſt wounds. I have not been able to aſcertain the ſpecific time (if ſuch there be) after the infliction of a wound, when we may pretty certainly ſay there is no danger of this ſpaſm appearing; nor what is the uſual time of its coming on: but ſhall make it my buſineſs to attend to both theſe points.

*Gibraltar,*
*November* 3, 1792.

III. *An*

